# Sexual Expression in Old Age: A Qualitative Study

**DOI:** 10.1093/geroni/igaa057.1004

**Published:** 2020-12-16

**Authors:** Sofia von Humboldt, José Alberto Ribeiro-Gonçalves, Andrea Costa

**Affiliations:** Instituto Superior de Psicologia Aplicada (ISPA), Lisbon, Portugal

## Abstract

Objective: This study aims to analyze how older adults express themselves sexually. For this purpose, a qualitative research was carried out, which analyzes their perspectives at a cross-national level. Methods: Four hundred and ninety five older participants aged 65 to 98 years, were interviewed. Participants were of three different nationalities and lived in the community. All the interviews went through the process of verbatim transcription and subsequent content analysis. Results: Results of content analysis produced nine themes: Tender and care (k = .91, p < .01); altruism and gratitude (k = .81, p < .01); attractiveness (k = 94, p < .01); positive communication (k = .89, p < .01); sexual activity (k = .88, p < .01); good health and physical condition (k = .96, p < .01); supportive relationship (k = .84, p < .01); eroticism (k = .94, p < .01); and feeling active and alive (k = .92, p < .01). Conclusions: This study stressed different ways on expressing sexuality in old age and underlined the importance of tender and care and eroticism for older adults who are sexually active. Keywords: Content analysis; older adults; qualitative study; sexual expression.

